# Abnormal Microvascular Architecture, Fibrosis, and Pericyte Characteristics in the Calf Muscle of Peripheral Artery Disease Patients with Claudication and Critical Limb Ischemia

**DOI:** 10.3390/jcm9082575

**Published:** 2020-08-08

**Authors:** Constance J. Mietus, Timothy J. Lackner, Petros S. Karvelis, Gregory T. Willcockson, Christina M. Shields, Nicholas G. Lambert, Panagiotis Koutakis, Matthew A. Fuglestad, Hernan Hernandez, Gleb R. Haynatzki, Julian K. S. Kim, Holly K. DeSpiegelaere, Iraklis I. Pipinos, George P. Casale

**Affiliations:** 1 Department of Surgery, University of Nebraska Medical Center, Omaha, NE 68198-3280, USA; constance.mietus@unmc.edu (C.J.M.); tim.lackner@unmc.edu (T.J.L.); pkarvelis@gmail.com (P.S.K.); gregory.willcockson@unmc.edu (G.T.W.); christina.shields@unmc.edu (C.M.S.); nicholas.lambert@unmc.edu (N.G.L.); pkoutakis@fsu.edu (P.K.); matthew.fuglestad@unmc.edu (M.A.F.); hhernand@unmc.edu (H.H.); kkim@unmc.edu (J.K.S.K.); 2 Department of Nutrition, Food and Exercise Sciences, Florida State University, Tallahassee, FL 32306, USA; 3 Department of Biostatistics, University of Nebraska Medical Center, Omaha, NE 68198-4375, USA; ghaynatzki@unmc.edu; 4 Department of Surgery and VA Research Service, VA Nebraska-Western Iowa Health Care System, Omaha, NE 68105-1873, USA; holly.despiegelaere@va.gov

**Keywords:** microvascular pathology, αSMA+ pericytes, basement membrane thickening, fibrosis

## Abstract

Work from our laboratory documents pathological events, including myofiber oxidative damage and degeneration, myofibrosis, micro-vessel (diameter = 50–150 μm) remodeling, and collagenous investment of terminal micro-vessels (diameter ≤ 15 µm) in the calf muscle of patients with Peripheral Artery Disease (PAD). In this study, we evaluate the hypothesis that the vascular pathology associated with the legs of PAD patients encompasses pathologic changes to the smallest micro-vessels in calf muscle. Biopsies were collected from the calf muscle of control subjects and patients with Fontaine Stage II and Stage IV PAD. Slide specimens were evaluated by Quantitative Multi-Spectral and Fluorescence Microscopy. Inter-myofiber collagen, stained with Masson Trichrome (MT), was increased in Stage II patients, and more substantially in Stage IV patients in association with collagenous thickening of terminal micro-vessel walls. Evaluation of the Basement Membrane (BM) of these vessels reveals increased thickness in Stage II patients, and increased thickness, diameter, and Collagen I deposition in Stage IV patients. Coverage of these micro-vessels with pericytes, key contributors to fibrosis and BM remodeling, was increased in Stage II patients, and was greatest in Stage IV patients. Vascular pathology of the legs of PAD patients extends beyond atherosclerotic main inflow arteries and affects the entire vascular tree—including the smallest micro-vessels.

## 1. Introduction

Peripheral artery disease (PAD) is a severe atherosclerotic condition, primarily of the elderly, which afflicts more than 200 million individuals worldwide [1]. PAD patients carry an increased risk of cardiovascular morbidity and mortality [2]. Patients with PAD have decreased calf muscle strength and exhibit a wide range of lower leg symptoms and signs [3,4]. Approximately one-third of PAD patients experience intermittent claudication, identified as walking cessation caused by exertional calf pain that resolves with several minutes of rest (Fontaine Stage II) [5,6]. Patients with more advanced disease experience pain at rest (Fontaine Stage III) [6,7,8] or tissue loss and gangrene (Fontaine Stage IV) [6,7]. Restriction of blood flow by large-artery plaques, does not fully account for lower extremity dysfunction in PAD [5,9,10]. An early study of the calf muscle of PAD patients with intermittent claudication, documented pathological changes, including myofiber denervation, decreased cross-sectional area of Type II myofibers and reduced cross-sectional muscle area [10].

Pathological events in the lower-extremity muscles of PAD patients include the presence of central nuclei in the myofibers (an index of fiber regeneration), structural changes in organelles, phagocytic cells in the more damaged myofibers, and myofiber degeneration [9]. Morphometry of myofibers in calf muscle has identified systematic changes with disease progression [11,12] that predict changes in calf muscle strength, six-minute walk and maximum treadmill walking time [13]. Myofiber degeneration identified as reduced cross-sectional area [11,14] and replacement with fibrotic tissue [15] is linked to oxidative damage in the form of 4-hydroxy-2-nonenal and carbonyl adducts [14]. Fibrosis is associated with hyperplastic, Transforming Growth Factor-β1 (TGFβ1) laden vascular smooth muscle cells in enlarged media of arterioles and venules and dense collagenous investment of micro-vessels [15].

In this study, we extend our observation of collagenous investment of micro-vessels in the calf muscle of PAD patients [15] to quantitative analyses of basement membrane (BM) architecture and collagen deposition, as well as pericyte coverage of micro-vessels, and test the hypothesis that these measures of micro-vessel pathology increase with disease progression.

## 2. Experimental Section

### 2.1. Human Subjects

The study protocol was approved by the University of Nebraska Medical Center and the Veterans Affairs Nebraska-Western Iowa Institutional Review Boards. All subjects gave informed consent.

#### 2.1.1. Control Group

We recruited 14 control patients who were healthy volunteers or had undergone vascular operations for abdominal aortic aneurysm repair, carotid endarterectomy, or bypass of a popliteal aneurysm. Control patients led sedentary lifestyles and had no history of PAD symptoms. Control patients had normal blood flow to their lower limbs as indicated by normal lower extremity pulses at examination and normal Ankle Brachial Index (ABI) at rest and after stress.

#### 2.1.2. PAD Groups

We recruited 15 patients with claudication (Fontaine Stage II) and 16 patients presenting with tissue loss (Fontaine Stage IV) who were scheduled for lower extremity operations for symptomatic PAD. Medical history, physical examination, decreased ABI (<0.9), and computerized or standard arteriography that revealed stenotic and/or occluded arteries supplying the lower extremities were evaluated to establish the diagnosis for each PAD patient. Stage II PAD patients presented with intermittent claudication, but no rest pain or tissue loss. Stage IV PAD patients presented with non-healing ulcers and/or gangrene.

### 2.2. Biopsy

Calf muscle samples weighing approximately 250 mg were obtained from the anteromedial aspect of the muscle belly, 10 cm distal to the tibial tuberosity. All biopsies were obtained with a 6 mm Bergstrom needle. Samples were placed immediately into cold Methacarn fixative, transferred at 48 h to cold 50% ethanol, and 5 to 10 days later embedded in paraffin.

### 2.3. Multi-Spectral Brightfield Microscopy: Quantification of Inter-Myofiber Collagen

#### 2.3.1. Specimen Staining

Paraffin-embedded calf muscle biopsies sectioned at four microns and mounted to glass slides were stained with the Thermo Scientific Richard-Allan Scientific Masson Trichrome staining kit (ThermoFisher cat# 22-110-648). Slides were deparaffinized in xylene and hydrated through a series of ethanol concentrations to water. Briefly, the slides were held in Bouin’s Fluid for 1 h at 56 °C and then rinsed with water at room temperature and stained with Weigerts Iron Hematoxylin according to the manufacturer’s specifications. Slides were rinsed with water and transferred to Biebrich Scarlet-Acid Fuchsin Solution for 3 to 3½ min at room temperature. After 3 min, slides were transferred in pairs to deionized water at 10 s intervals. After 30 s in water, the first pair was transferred to the phosphotungstic-phosphomolybdic acid solution with subsequent pairs transferred every 10 s. After 5 min, the first pair was transferred to aniline blue stain solution and remained there for 10½ min. The remaining pairs were transferred to the stain solution at 10 s intervals with the last pair staining for 10 min. The remaining steps followed the manufactures recommendations. Specimens were dehydrated through xylene and mounted with a cover slip, in permount (Fisher Catalog Number Sp15-500).

#### 2.3.2. Image Acquisition

Masson Trichrome stained slide specimens of calf muscle were viewed as bright field images with the Nuance MultiSpectral Imaging System (N-MSI-EX Model, software version 3.0.2) (AKOYA BIOSCIENCES, Marlborough, MA, USA) fitted to a Leica DMRXA2 microscope with a 20× objective in place. Five 20× fields of interest were selected such that muscle filled each field. The system was set to produce an absorbance profile from 450 nm to 700 nm in 10 nm increments, at each pixel of the two-dimensional field. The absorbance spectra for collagen, myofibers and nuclei were input to the system software which converted each pixel to the quantity of material corresponding to each spectrum at the pixel. In this way, the system produced separate two-dimensional greyscale (12-bit) images of collagen, myofibers and nuclei. Greyscale images were imported into the Image Pro^®^ Premiere 9.3 (Media Cybernetics, Warrendale, PA, USA) environment for quantification of the intensities (greyscale units; gsu) and areas (micron^2^) of the inter-myofiber collagen events exclusive of large vessel collagen. Collagen abundance (gsu) was computed as the sum of the products (gsu∙micron^2^) of mean pixel intensity and event area divided by the total area (micron^2^) of myofibers plus total area of inter-myofiber collagen, per microscopic field. The average of five fields per slide specimen was taken as the collagen abundance per patient.

### 2.4. Quantitative Fluorescence Microscopy

#### 2.4.1. General

Paraffin-embedded biopsies were sectioned at 4 μm and mounted to glass slides. Slide specimens were deparaffinized with xylene and rehydrated via a series of ethanol washes and distilled water. The rehydrated slide specimens were heated at 92 °C in either 10 mM citrate buffer (pH 6.0) or 10 mM tris with EDTA buffer (pH 9.0) for 15 min and then allowed to cool at room temperature for 20 min. Subsequently, slide specimens were washed in Super-Sensitive Wash buffer (BioGenex Laboratories, Freemont, CA, USA) for 30 min at room temperature, followed by a 10 min wash in Dulbecco’s Phosphate Buffered Saline. Then, the specimens were transferred to a programmable robotic autostainer (BioGenex i6000, BioGenex Laboratories) where they were washed with Super Sensitive Wash Buffer and blocked with normal sera. Primary antibodies and corresponding immunoglobulin isotypes were pipetted manually onto the appropriate slides and held overnight at 4 °C. On the following day, slide specimens were processed at room temperature with the programmable autostainer. They were washed with Super-Sensitive Wash buffer, labeled with secondary Ab for one hour and washed with Dulbecco’s Phosphate Buffered Saline. The specimens were mounted in Prolong Diamond Mounting Media that contained nuclear label (DAPI: 4′,6-diamidino-2-phenylindole; Invitrogen, Carlsbad, CA, USA). For each patient specimen, duplicate slides were treated with primary Ab, and a third slide was treated with corresponding immunoglobulin isotype.

Gray-scale (12-bit) images (1344 × 1044 pixels) were captured with a widefield, epifluorescence microscope (Leica DMRXA2; North Central Instruments, Plymouth, MN, USA) (10× objective, 0.5128 μm/pixel or 40× objective, 0.129 μm/pixel), a B/W CCD camera (Orca ER C4742-95; Hamamatsu Photonics, Bridgewater, NJ, USA) and HCImage software (32-bit version, 4.2.5; Hamamatsu Photonics). Captured images were coded so that analysis was blinded to the patient group.

#### 2.4.2. Quantification of Micro-Vessel Associated Collagen I (Principal Collagen of the Extracellular Matrix) and Collagen IV (Marker of the Basement Membrane)

Specimens were heated in citrate buffer at 92 °C for 15 min, cooled at room temperature, blocked with 10% goat serum (cat# 500622, Life Technologies, Carlsbad, CA, USA) and then treated overnight at 4 °C with rabbit polyclonal anti-Collagen I Ab (10 μg/mL, cat# NB600-408, Novus Biologicals, Centennial, CO, USA) or mouse monoclonal anti-Collagen IV Ab (10 μg/mL, clone M3F7, Developmental Studies Hybridoma Bank, Iowa City, IA, USA). Isotype control specimens were treated with rabbit polyclonal IgG (10 μg/mL, cat#NBP2-24891, Novus Biologicals) or mouse IgG1κ (10 μg/mL, ref# 14-4714-85 Ebioscience, San Diego, CA, USA). Subsequently, the specimens were labeled with a mixture of goat anti-mouse IgG-Alexa Fluor 647 (2 μg/mL, cat# A21236, Molecular Probes, Eugene, OR, USA) or goat anti-rabbit IgG-Alexa Fluor 647 (2 μg/mL, cat# A21429, Molecular Probes) and Wheat Germ Agglutinin (WGA)-Alexa Fluor 488 (5 μg/mL, cat# W11261, Molecular Probes). For evaluation of co-localization of Collagen I and Collagen IV, specimens were labeled with a mixture rabbit anti-Collagen I Ab and mouse anti-Collagen IV Ab and subsequently, with a mixture of goat anti-mouse IgG-Alexa Fluor 647 and goat anti-rabbit IgG-Alexa Fluor 555.

HCImage was programmed for automated acquisition of the fluorescence signals from the slide-mounted muscle specimens. The entirety of each slide specimen was captured in the 647 nm channel (Collagen I or Collagen IV) with a 10× objective, producing 70–150 microscope frames (10× fields). The acquired fields were montaged into one image representing the entire slide specimen. Twelve regions of interest (ROIs) were identified from a review of the montage, and the ROIs were located on the original slide specimen. For micro-vessel collagen measurements, four contiguous frames (2 × 2) in each ROI were captured with a 40× objective, in three fluorescence channels corresponding to nuclei (DAPI: 358 nm excitation max and 461 nm emission max), cell membrane and extracellular matrix (WGA-Alexa Fluor 488: 488 nm excitation max and 520 nm emission max) and Collagen I (Alexa Fluor 647: 650 nm excitation max and 668 nm emission max) or Collagen IV (Alexa Fluor 647: 650 nm excitation max and 668 nm emission max). Fluorescence intensities produced by Ab treatment were corrected for fluorescence intensities produced by the corresponding isotype controls.

**Figure 1:**
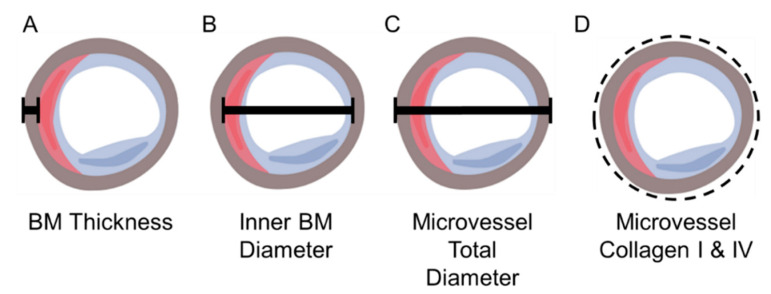
Schematic Representation of Micro-vessel Architecture. The drawing depicts a micro-vessel with endothelial cell (blue), pericyte (red) and basement membrane (BM) (brown). The black bars identify micro-vessel measurements: (**A**) BM thickness, (**B**) BM inner diameter and (**C**) micro-vessel overall diameter. The dashed line (**D**) identifies Area of Interest (AOI) placed around each micro-vessel.

The labeled collagen ring associated with each micro-vessel was partitioned in the Image Pro^®^ Plus software environment and both ring area (μm^2^) and mean fluorescence intensity (greyscale units; gsu) were captured. We placed a circular area of interest (AOI) around each collagen ring to restrict partitioning to the collagen ring (Figure 1D). Collagen abundance for each micro-vessel was calculated as the product of ring area and mean fluorescence intensity (gsu·μm^2^) corrected for isotype background fluorescence. Mean collagen abundance per micro-vessel was determined for the 12 ROIs of each slide specimen, and the abundance per patient was determined as the average of two slides.

#### 2.4.3. Measurement of Micro-Vessel Basement Membrane Thickness, Inner Diameter, and Overall Diameter

**Figure 2:**
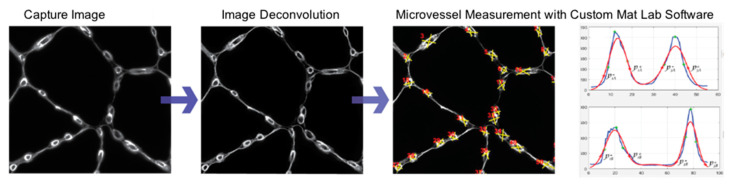
Measurements of Micro-vessel Architecture. Slide specimens labeled with anti-Collagen IV Ab were imaged with a 40× objective and deconvoluted with AutoQuant^®^ software for reduced light scatter and improved micro-vessel delineation. For each micro-vessel (overall diameter ≤15μm), two perpendicular line profiles of Collagen IV fluorescence intensity were generated with a custom MatLab program. Gaussian distributions (red) were fitted to the raw fluorescence intensity data (blue). The thickness of the Collagen IV ring (basement membrane) for each micro-vessel was determined as the average distance at the 95% confidence interval for each of the Gaussian curves. The inner diameter of the basement membrane was determined as the mean of the distances between the pairs of Gaussian curves at the locations of their 95% confidence intervals.

Greyscale images from 12 ROIs of each slide specimen labeled with anti-Collagen IV Ab and captured with a 40× objective as a montage of 2 × 2 contiguous microscope fields per ROI were imported into the Image Pro^®^ Plus software (Media Cybernetics, Warrendale, PA, USA) environment and corrected for isotype fluorescence intensities. Subsequently, the corrected montages were imported into the AutoQuant^®^ software (Media Cybernetics) environment for deconvolution that improved image resolution and contrast. Measurements of micro-vessel architecture in the deconvoluted images were implemented with a custom Matlab program (MathWorks, Inc., Natick, MA, USA). Briefly, two perpendicular lines were drawn such that they transected a micro-vessel. For vessels that were not perfectly circular, one of the perpendicular lines transected the vessel at its longest diameter. The program extracted and plotted Collagen IV fluorescence intensities along each line and fitted the raw fluorescence data to Gaussian distributions (Figure 2). The thickness of the Collagen IV ring (basement membrane) for each micro-vessel was determined as the average distance at the 95% confidence interval of each Gaussian curve. The inner diameter of the basement membrane was determined as the mean of the distances between the pairs of Gaussian curves at the locations of their 95% confidence intervals. Total micro-vessel diameter was calculated as the mean inner basement membrane diameter plus twice the mean basement membrane thickness for each micro-vessel (Figure 2). To ensure uniform cross-sectional measurements, we measured only micro-vessels whose total micro-vessel diameter at each of the perpendicular lines exhibited a ratio of 0.75 to 1.00 (corresponding to a perfect circle). Approximately 50–250 micro-vessels were assessed per slide specimen. The number of micro-vessels evaluated per ROI varied, reflecting the variation of the micro-vessel density per specimen.

#### 2.4.4. Identification of Micro-Vessel Associated Pericytes

We identified pericytes as α-smooth muscle actin (αSMA) positive cells (1) located abluminal to CD31 positive endothelial cells lining micro-vessels (overall diameter ≤ 15 microns) and (2) intimately associated with the Collagen IV positive basement membrane of these micro-vessels.

Slide specimens were blocked with 10% donkey serum (cat# ab7475, Abcam, Cambridge, MA, USA) and then treated with a mixture of mouse monoclonal anti-collagen IV Ab (10 μg/mL, clone M3F7, Developmental Studies Hybridoma Bank, Iowa City, IA, USA), rabbit polyclonal anti-αSMA Ab (1 μg/mL, cat# ab5694, Abcam, Cambridge, MA, USA) and sheep polyclonal anti-CD31 Ab (3.5 μg/mL, cat# AF806-SP, R&D Systems, Minneapolis, MN, USA). Isotype control specimens were treated with a mixture of mouse IgG1κ, normal rabbit polyclonal IgG (1 μg/mL, cat# AB-105-C, R&D System, Minneapolis, MN, USA), and normal sheep polyclonal IgG (3.5 μg/mL, cat# 5-001-A, R&D Systems, Minneapolis, MN, USA). Subsequently, the slides were treated with a mixture of donkey anti-mouse IgG-Alexa Fluor 488 Ab (2 μg/mL, cat# A21202, Molecular Probes, Eugene, OR, USA), donkey anti-rabbit IgG-Alexa Fluor 555 Ab (2 μg/mL, cat# A31572, Molecular Probes, Eugene, OR, USA), and donkey anti-sheep IgG-Alexa Fluor 647 Ab (2 μg/mL, cat# A21448, Molecular Probes, Eugene, OR, USA).

Five microscope fields per duplicate slide specimen were captured with a 40× objective, in four fluorescence channels corresponding to (1) DAPI (nuclei), (2) Alexa Fluor 488 (Collagen IV) with an excitation maximum at 495 nm and emission maximum at 519 nm, (3) Alexa Fluor 555 (αSMA), and (4) Alexa Fluor 647 (CD31). Fluorescence intensities produced by Ab treatment were corrected for fluorescence intensities produced by the corresponding isotype controls.

### 2.5. Micro-Vessel Density

Greyscale images obtained from Collagen I and IV labeling were transferred into the Image Pro^®^ Plus environment. Collagen IV label uniformly identified micro-vessel structure and was used to create partitions that yielded accurate vessel identification. The number of micro-vessels and total tissue area were determined. A ratio of micro-vessels per total tissue area was calculated to determine micro-vessel density per each ROI assessed for micro-vessel architecture.

### 2.6. Statistical Analysis

Demographic variables were evaluated for significant differences across study groups (Fontaine Stage II and Stage IV PAD patients and controls) by the non-parametric Kruskal-Wallis Test, for continuous variables, or by the Yates’ Continuity Corrected Chi-Square Test, for categorical variables. All other between-group differences were evaluated by the two-tailed Student’s *t*-test for normally distributed data and by the non-parametric Mann-Whitney U test or randomization test for data that were not distributed normally. Selected clinical parameters were analyzed as covariates with a General Linear Model for fixed factors. All statistical analyses were completed with NCSS 2020 Statistical Software (NCSS, LLC. Kaysville, UT, USA).

## 3. Results

### 3.1. Patient Demographics

The number of subjects in each cohort (Control *N* = 14; Stage II PAD *N* = 15; Stage IV PAD *N* = 16) was limited by the labor-intensive measurements of micro-vessels in slide specimens of calf muscle. Previous studies of micro-vessel structure in lower leg muscles of PAD patients and control subjects documented significant differences with 8 to 14 subjects in each cohort; consequently, the number of subjects in each of our cohorts was considered adequate for this study. By design, mean ABI of controls was significantly different from both Stage II and Stage IV PAD patients (Table 1). It is of interest that ABI and prevalence of Diabetes Mellitus (DM), Renal Insufficiency (RI), and Family history of cardiovascular disease (FH) of Stage IV PAD patients differed significantly from both Stage II PAD patients and control subjects. These variables were treated as potential covariates in subsequent analyses.

### 3.2. Fibrosis Is Increased in Calf Muscle of Patients with PAD

**Figure 3:**
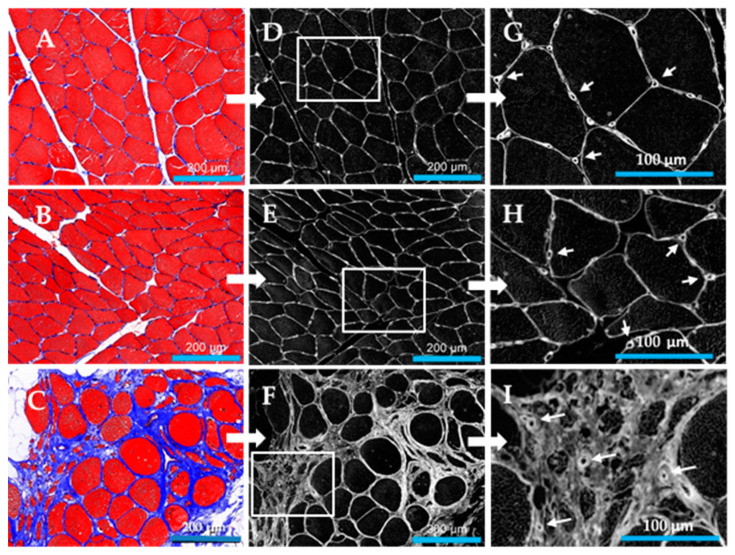
Inter-myofiber collagen deposition in the calf muscle of control subjects and patients with peripheral artery disease (PAD). Slide specimens of calf muscle biopsies fixed and then embedded in paraffin were sectioned at four microns and stained with Masson Trichrome (MT). Inter-myofiber collagen staining (**Blue**) in control calf muscle (Panel (**A**)) (*N* = 14) is uniform among closely associated myofibers (**RED**) of relatively homogeneous polygonal shape and cross-sectional area. Collagen staining is increased in PAD Stage II (Panel (**B**)) (*N* = 15) and Stage IV (Panel (**C**)) (*N* = 15) with the latter presenting remarkable departures in myofiber geometry and collagen deposition compared to both control and Stage II muscle. Stage IV calf muscle exhibited many small degenerating and fragmented myofibers embedded in a large fibrotic matrix, as well as enlarged rounded myofibers, likely necrotic, surrounded by more abundant collagen. Greyscale images of deposited collagen (Panels **D**–**F**) were extracted from Multi-Spectral images of slide specimens stained with MT and captured with the Nuance System (20× objective). A magnified region (Panels **G**–**I**) of each greyscale image revealed well-defined collagenous rings around the micro-vessels (**Arrows**). Scale bars (Panels **A**–**F**) represent 200 microns.

**Figure 4:**
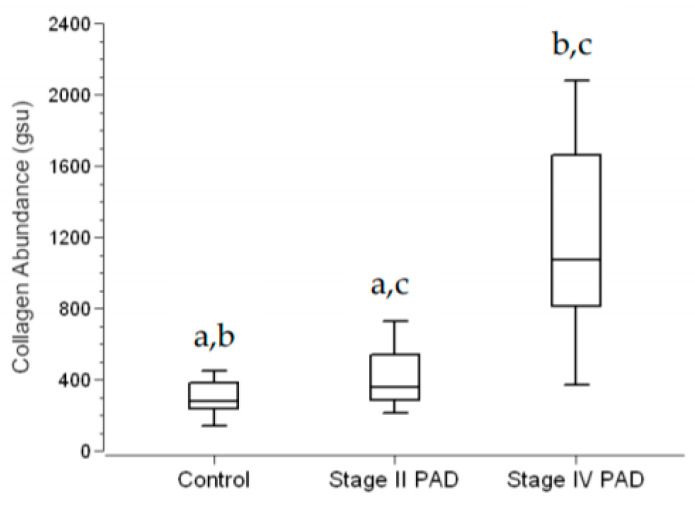
Inter-myofiber Collagen Abundance in Calf Muscle of Control Subjects and Patients with Peripheral Artery Disease (PAD). Calf muscle biopsies were taken from control subjects (*N* = 14) and patients with Stage II (*N* = 15) and Stage IV PAD (*N* = 15), embedded in paraffin, sectioned at four microns, and stained with Masson Trichrome (MT). Microscopic images were captured with a Multi-Spectral imaging system. Quantitative greyscale images of inter-myofiber collagen were extracted from the Multi-Spectral images and quantified in the Image Pro^®^ Premier environment. Collagen abundance (gsu) was computed as the sum of the products (gsu∙micron2) of mean pixel intensity and event area divided by the total area (micron2) of myofibers plus total area of inter-myofiber collagen, per microscopic field. The average of five fields per slide specimen was taken as the collagen abundance per patient. Data are presented as Fence Box Plots. Statistical significance was determined with the non-parametric randomization test. a—Significantly different at *p* = 0.041. b—Significantly different at *p* < 0.001 c—Significantly different at *p* < 0.001

Fibrosis in calf muscle was determined as collagen abundance, from measurements of inter-myofiber collagen exclusive of large vessel collagen. In control specimens (*N* = 14), collagen was uniformly distributed between closely associated myofibers of relatively homogeneous polygonal shape and cross-sectional area (Figure 3A). Stage II PAD specimens (*N* = 1 5) were characterized by increased variation in myofiber shape, reduced cross-sectional area and the appearance of more deeply stained inter-myofiber collagen (Figure 3B). Both myofiber geometry and inter-myofiber collagen deposition in Stage IV specimens (*N* = 15) (Figure 3C) presented remarkable departures from these features of control and Stage II specimens, exhibiting regions of degenerating, fragmented myofibers embedded in a fibrotic matrix, as well as large swollen fibers, likely undergoing necrosis, with abundant inter-myofiber collagen. Inter-myofiber collagen abundances in all specimens were determined from greyscale images (illustrated in Figure 3D–F) extracted from Multi-Spectral images of Masson Trichome stained specimens. Collagen abundances are presented as mean ± s.e.m. for each study group. Stage IV collagen abundance (1114 ± 130 gsu) was nearly three to four times greater than collagen abundances of Stage II patients (409 ± 39 gsu at *p* < 0.001) and control subjects (306 ± 29 gsu at *p* < 0.001). Collagen abundance of Stage II patients was greater than that of control subjects at *p* = 0.041 (Figure 4). Notably, micro-vessels were circumscribed with well-defined collagen rings (Figure 3G–I) that were larger and exhibited increased wall thickness, in Stage IV PAD patients.

Since the prevalence of DM, RI and FH were greater among Stage IV PAD patients (Table 1), these clinical parameters were evaluated as covariates in a General Linear Model for fixed factors (Group: Control, Stage II and Stage IV). For response variable Collagen Abundance, the predictive value (adjusted *R*^2^) of the model was 0.545 (*p* < 0.001) where Group (*p* < 0.001) was a significant predictor and DM (*p* = 0.367), RI (*p* = 0.613), and FH (0.750) were not significant predictors.

### 3.3. Micro-Vessel Basement Membrane Architecture Is Altered in Calf Muscle of Patients with PAD

**Figure 5:**
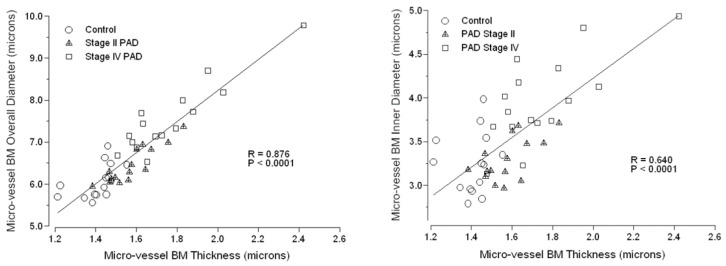
Dimensions of the architectural features of the micro-vessel basement membrane (BM) are highly coordinated across control subjects and PAD patients. BMs of calf muscle biopsy specimens were labeled with anti-Collagen IV antibody, and then micro-vessel BM architectural features, including thickness, inner diameter and overall diameter were measured. Measurements were obtained for approximately 50 to 200 micro-vessels per biopsy specimen of control subjects (*N* = 14) and patients with Stage II (*N* = 15) and Stage IV (*N* = 16) PAD. Means per patient are plotted. Correlations were determined by Spearman Rho.

Masson Trichrome labeling of collagen in calf muscle specimens of control subjects and patients with PAD revealed well-defined ring structures delineating calf muscle micro-vessels of all three study groups (Figure 3). The high definition and location of these ring structures suggested that they represented basement membrane which appeared to be greater in diameter and wall thickness in Stage IV patients compared to control subjects and Stage II PAD patients. To investigate the basement membrane architecture of micro-vessels, we labeled BMs of calf muscle slide specimens with anti-Collagen IV antibody and measured (μm) BM thickness and inner and overall BM diameter (Table 2). All three parameters of BM architecture were increased uniformly by 23% to 24% (*p* < 0.001) in micro-vessels of calf muscle specimens from patients with Stage IV PAD compared to control subjects. Compared to controls, BM thickness in micro-vessels of Stage II patients was increased by 12% (*p* < 0.001), inner diameter was not different and overall diameter was increased by 6% but did not reach statistical significance. The uniform increase of 23% to 24% in all BM architectural parameters in micro-vessels of Stage IV calf muscle compared to control muscle suggested that the dimensions of these BM parameters are coordinated. This was supported by analysis of the correlations of BM overall diameter (*R* = 0.876; *p* = 0.0001) and BM inner diameter (*R* = 0.640; *p* = 0.0001) and with BM thickness across all control subjects and PAD patients (Figure 5).

Since the prevalence of DM, RI and FH were greater among Stage IV PAD patients (Table 1), these clinical parameters were evaluated as covariates in a General Linear Model for fixed factors (Group: Control, Stage II and Stage IV). For response variable BM Thickness, the predictive value (adjusted *R*^2^) of the model was 0.359 (*p* < 0.001) where Group (*p* < 0.001) was a significant predictor and DM (*p* = 0.960), RI (*p* = 0.741), and FH (0.543) were not significant predictors. For response variable BM Inner Diameter, the predictive value (adjusted *R*^2^) of the model was 0.407 (*p* < 0.001) where Group (*p* < 0.001) was a significant predictor and DM (*p* = 0.288), RI (*p* = 0.344), and FH (0.962) were not significant predictors. For response variable BM Overall Diameter, the predictive value (adjusted *R*^2^) of the model was 0.432 (*p* < 0.001) where Group (*p* < 0.0001) was a significant predictor and DM (*p* = 0.542), RI (*p* = 0.460), and FH (0.728) were not significant predictors.

### 3.4. The Abundance of Collagen IV in Calf Muscle Micro-Vessels across Control Subjects and PAD Patients Reflects the Architectural Measurements of Micro-Vessel Basement Membranes (BM)

**Figure 6:**
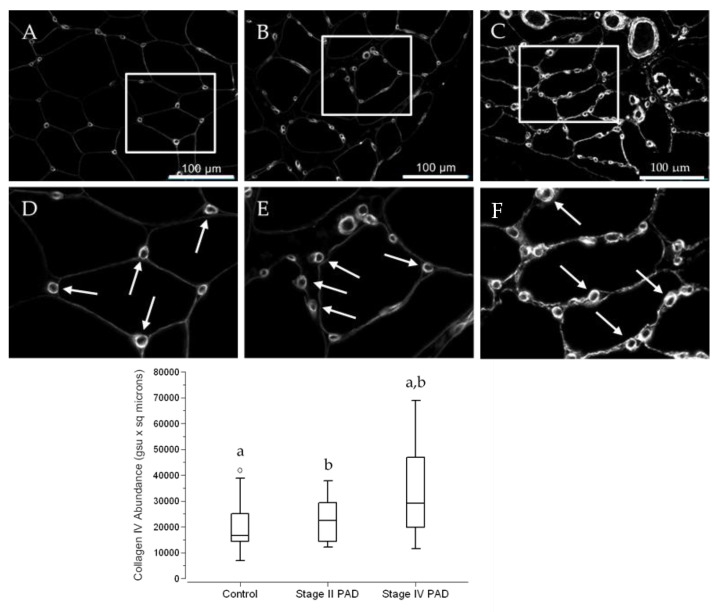
Collagen IV deposition presents as intensely labeled BM rings that delineate micro-vessels in the calf muscle of control subjects and PAD patients. Slide specimens of biopsies from calf muscle of control subjects (**A**) (*N* = 14) and patients with Stage II (**B**) (*N* = 15) and Stage IV (**C**) (*N* = 16) PAD were labeled with anti-Collagen IV antibody, and fluorescence images were captured with a widefield microscope (40× objective). Selected regions of the same dimensions in Panels (**A**–**C**) were enlarged (Panels (**D**–**F**)), providing a more detailed view of the micro-vessel labeling (**Arrows**). Both inner and overall diameters of the BM rings appear to be larger and more intense in the specimen from the patient with Stage IV disease, compared to specimens from the control subject and the patient with Stage II disease. Scale bars represent 100 μm. Median Collagen IV abundances per micro-vessel were determined by Quantitative Fluorescence Microscopy, computed as the product of ring area and mean labeling intensity, and are presented as Fence Box Plots. Collagen IV abundance was significantly increased in patients with Stage IV PAD compared to control subjects and Stage II patients. Data were analyzed by the randomization test where: a—significantly different at *p* = 0.029. b—significantly different at *p* = 0.044.

Slide specimens of calf muscle biopsies collected from control subjects and patients with PAD were labeled with the antibody specific for Collagen IV, a marker for BM. For both control subjects and PAD patients, Collagen IV labeling delineated individual myofibers and presented as well-defined rings of intense labeling associated with the micro-vessels (Figure 6A–C). Apart from control specimens, micro-vessel Collagen IV abundance was somewhat heterogeneous, but generally was greater in specimens from PAD patients with Stage IV disease compared to patients with Stage II disease (Figure 6D–F). Using Quantitative Fluorescence Microscopy, we quantified micro-vessel Collagen IV abundance in slide specimens labeled with anti-Collagen IV antibody (Figure 6; Fence Box Plot). For each micro-vessel, abundance was determined as the product of mean fluorescence intensity (pixel) and area occupied by Collagen IV. Medians (M) and interquartile ranges (IQR) were 16,670 gsu·μm^2^, 12,778 gsu·μm^2^; 22,378 μm^2^, 14,860 μm^2^; and 29,165 μm^2^, 27,286 μm^2^ for control, Stage II PAD and Stage IV PAD specimens, respectively. Collagen IV abundance of Stage IV patients was 75% greater than that of control subjects (*p* = 0.029) and 30% greater than Stage II patients (*p* = 0.042). Abundances in Stage II compared to control specimens was increased by 34% but were not statistically significant (*p* = 0.581). Micro-vessel counts per unit area of slide specimen increased with advancing stage of the disease and reflected increased Collagen IV accumulation in micro-vessels with advancing disease, suggesting a coordinated response to the intermittent ischemia (hypoxia) of PAD. Micro-vessel counts in the calf muscle of Stage IV patients (5.9 micro-vessels per 10^4^ µm^2^) were 23% greater (*p* = 0.011) than controls (4.8 micro-vessels per 10^4^ μm^2^) and counts for Stage II patients (5.6 micro-vessels per 10^4^ μm^2^) were 17% greater (*p* = 0.017) than controls. The difference between Stage IV and Stage II patients did not reach significance (*p* = 0.256).

DM, RI and FH were evaluated as covariates in a General Linear Model for fixed factors (Group: Control, Stage II and Stage IV patients). For response variable Collagen IV abundance, the predictive value (adjusted *R*^2^) of the model was 0.103 (*p* = 0.099) where Group (*p* = 0.099) was not a significant predictor and DM (*p* = 0.965), RI (*p* = 0.513), and FH (*p* = 0.270) were not significant predictors. With DM, RI and FH excluded, the model had a predictive value of 0.120 (*p* = 0.026) and Group (*p* = 0.0258) was a significant predictor. For response variable Micro-vessel Density, the predictive value (adjusted *R*^2^) of the model was 0.150 (*p* = 0.043) where Group (*p* = 0.010) was a significant predictor and DM (*p* = 0.128), RI (*p* = 0.547), and FH (*p* = 0.916) were not significant predictors.

### 3.5. The Abundance of Collagen I, a Principal Contributor to Fibrosis, Is Increased in Terminal Micro-Vessels in Calf Muscle of PAD Patients with More Advanced Fibrosis

**Figure 7:**
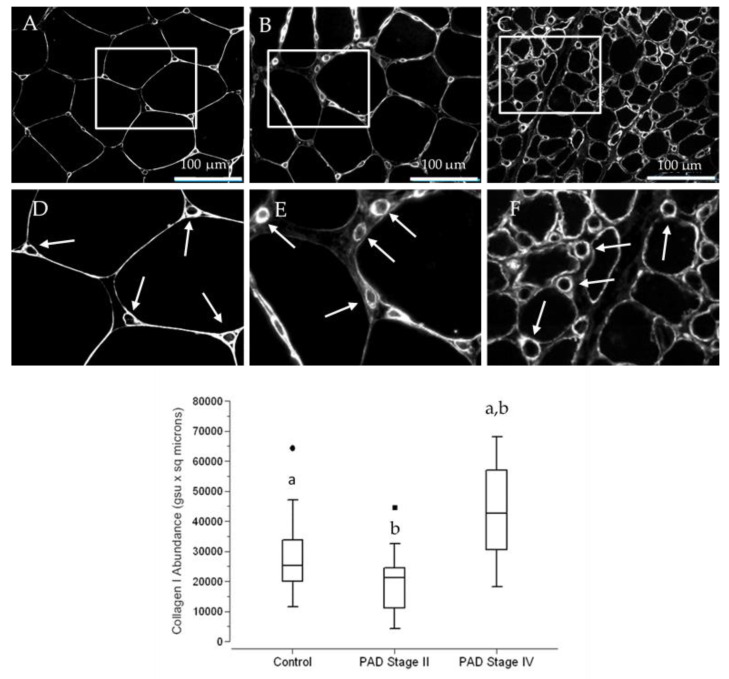
Collagen I deposition presents as intensely labeled rings that delineate micro-vessels in the calf muscle of control subjects and PAD patients. Slide specimens of biopsies from calf muscle of control subjects (**A**) (*N* = 14) and patients with Stage II (**B**) (*N* = 15) and Stage IV (**C**) (*N* = 16) PAD were labeled with anti-Collagen I antibody, and fluorescence images were captured with a widefield microscope (40× objective). Selected regions in Panels (**A**–**C**) were enlarged (Panels (**D**–**F**)), providing a more detailed view of micro-vessel labeling (**Arrows**). The inner and overall diameter of the micro-vessel rings appears to be larger and more intense in the specimen from the patient with Stage IV disease, compared to specimens from the control subject and the patient with Stage II disease. Scale bars = 100 µm. Median Collagen I abundances per micro-vessel were determined by Quantitative Fluorescence Microscopy, computed as the product of ring area and mean labeling intensity, and are presented as Fence Box Plots. Collagen I was significantly increased in patients with Stage IV PAD compared to control subjects and Stage II patients. Data were analyzed by the randomization test where: a—significantly different at *p* < 0.002. b—significantly different at *p* < 0.001

Collagen I production is increased substantially in models of pathological fibrosis [16], in association with increased expression of TGFβ1 [17]. Type I pericytes, micro-vessel subendothelial cells that produce Collagen I and have been implicated as the principal source of myofibroblasts in models of fibrosis [16]. In a previous study [15], our laboratory established the presence of hyperplastic VSMC, laden with TGFβ1, in small vessels (50 to 150 µm diameter) of the calf muscle of patients with PAD. Patients with Stage IV disease were characterized by advanced fibrosis. In this study, we evaluated the possibility of micro-vessel peri-endothelial deposition of Collagen I in the calf muscle of control subjects and patients with Stage II and Stage IV PAD. Slide specimens of calf muscle biopsies collected from control subjects and patients with PAD were labeled with anti-Collagen I antibody. Collagen I deposition presented as well-defined micro-vessel rings (Figure 7) that were generally larger in the calf muscle of Stage IV patients. Using Quantitative Fluorescence Microscopy, we quantified micro-vessel Collagen I abundance in slide specimens labeled with anti-Collagen I antibody (Figure 7; Fence Box Plot). For each micro-vessel, abundance was determined as the product of mean fluorescence intensity (mean pixel) and area occupied by Collagen I. Medians (M) and interquartile ranges (IQR) were 23,058 gsu·m^2^, 15,877 gsu·μm^2^; 21,272 gsu·μm^2^, 13,369 gsu·μm^2^; and 42,660 gsu·μm^2^, 26,412 gsu·μm^2^ for control subjects and Stage II and Stage IV PAD patients, respectively. In a randomization test, Collagen I abundance of Stage IV patients was greater than that of control subjects (*p* < 0.002) and Stage II patients (*p* < 0.001). Collagen I abundances in specimens of control subjects and Stage II PAD patients were not significantly different (*p* = 0.305).

DM, RI and FH were evaluated as covariates in a General Linear Model for fixed factors (Group: Control, Stage II and Stage IV patients). For Collagen I Abundance as response variable, the predictive value (adjusted *R*^2^) of the model was 0.316 (*p* < 0.002) where Group (*p* < 0.007) was a significant predictor and DM (*p* = 0.738), RI (*p* = 0.689), and FH (*p* = 0.987) were not significant predictors.

### 3.6. Collagen I and Collagen IV Were Co-Localized in the Basement Membrane of Calf Muscle Biopsies from Control Subjects and Patients with PAD

**Figure 8:**
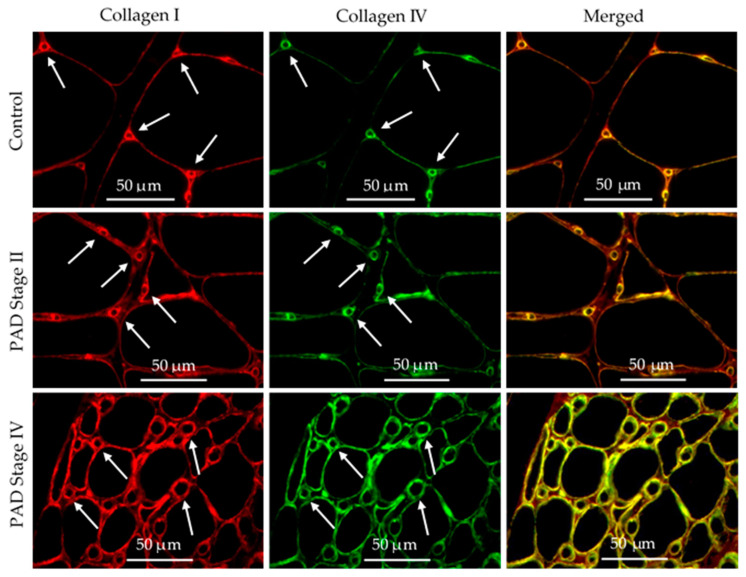
Co-localization of Collagen I and IV deposited as distinctive rings associated with micro-vessels in the calf muscle of control subjects and patients with PAD. Biopsy specimens embedded in paraffin were sectioned at four microns and mounted to glass slides. Slide specimens were labeled simultaneously with anti-Collagen I and anti-Collagen IV antibodies, and subsequently, with goat anti-rabbit and goat anti-mouse secondary antibodies (respectively) coupled with fluorophores. Images of each slide specimen were taken in separate fluorescence channels corresponding to each collagen label, with a widefield microscope (40× objective). Distinctive rings of Collagen I (**Red**) and Collagen IV (**Green**) delineate micro-vessels (**Arrows**) and are co-localized (**Yellow**) in the basement membrane. Myofibers are identified as large black areas delineated by both Collagen I and Collagen IV. Labeling of inter-myofiber Collagen I (**Red**) is readily apparent in the merged images of the Stage II and Stage IV specimens. Scale bars represent 50 microns.

The patterns of deposition of Collagen IV (Figure 6) and Collagen I (Figure 7) in micro-vessels of calf muscle biopsies of control subjects and patients with PAD are similar and suggest that both may be expressed in the basement membrane of the micro-vessels. To test this possibility, we evaluated co-localization of the fluorescence signals of these collagens in calf muscle micro-vessels. Slide specimens of calf muscle biopsies from control subjects and patients with PAD were labeled concurrently with a rabbit antibody specific for Collagen I and a mouse monoclonal antibody specific for Collagen IV. Subsequently, the specimens were treated with a mixture of AlexaFluor 555 conjugated goat anti-rabbit IgG and AlexaFluor 647 conjugated goat anti-mouse IgG. Each microscopic field (40× objective) was captured in two fluorescence channels corresponding to the secondary fluorescent antibodies. Fluorescence images of Collagen I deposition pseudo-colored red and images of Collagen IV deposition pseudo-colored green are presented as independent images and as merged images of control subjects, Stage II PAD patients and Stage IV PAD patients (Figure 8). Fibrosis in specimens of Stage II and Stage IV patients is evident as inter-myofiber labeling with anti-Collagen I antibody in both the separate and merged images. Fibrosis is more pronounced in the Stage IV specimens. The merged images demonstrate co-localization of the Collagen I and Collagen IV signals (yellow) in the basement membrane of micro-vessels in control, Stage II and Stage IV specimens (Figure 8).

### 3.7. Pericyte Coverage of Micro-Vessels in Calf Muscle Specimens Was Greater in Patients with Stage IV Disease Compared to Controls and Patients with Stage II Disease

**Figure 9:**
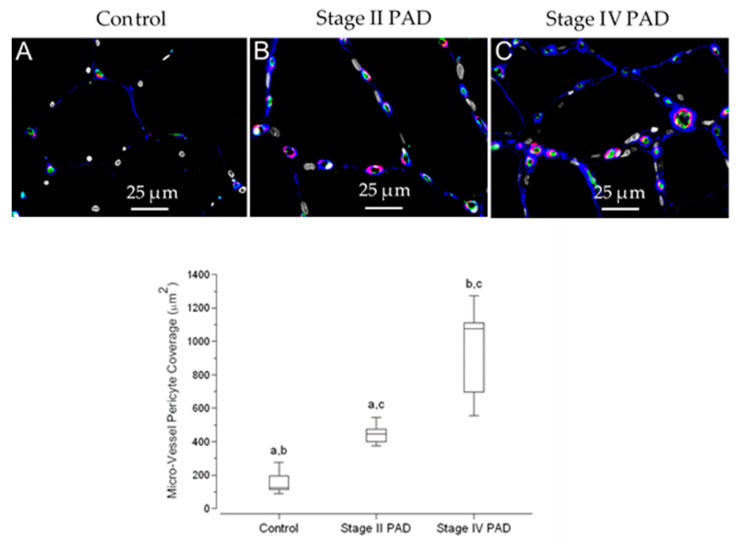
Micro-vessel pericyte coverage is increased in the calf muscle of patients with PAD. Slide specimens of calf muscle biopsies from control subjects (*N* = 5) and patients with Stage II (*N* = 5) and Stage IV (*N* = 5) PAD were labeled with antibodies specific for Collagen IV (**Blue**), αSmooth Muscle Actin (αSMA) (**Red**), endothelial cell CD-31 (**Green**) and nuclei (**Grey**/**White**). Fluorescence images were captured with a widefield microscope (40× objective). Pericyte segments were identified as αSMA positive labeling intimately associated with the micro-vessel endothelium. Micro-vessels of control muscle (**A**) had few pericyte segments, and these occupied very small areas. The number and size of pericyte labeling events increased in patients with Stage II PAD (**B**) and were greater in patients with Stage IV PAD (**C**). Scale bar = 25 μm. Pericyte coverage per microscopic field was determined by Quantitative Fluorescence Microscopy as the sum of the areas occupied by pericyte segments. We analyzed five 40× fields in duplicate slide specimens per patient and control. Coverage for controls and patients with Stage II and Stage IV disease is presented as Fence Box Plots, and differences were analyzed by the randomization test where: a—significantly different at *p* = 0.009; b—significantly different at *p* < 0.009; c—significantly different at *p* < 0.007.

Pericytes, along with endothelial cells, are required for basement membrane assembly and remodeling [18]. In the present study, we documented basement membrane remodeling in the calf muscle of patients with Stage II and Stage IV PAD. Late-stage disease is characterized by increased basement membrane thickening and diameter in association with fibrosis and greater abundances of basement membrane Collagen IV and Collagen I. Consequently, we evaluated pericyte coverage of micro-vessels in relation to progression of PAD. Slide specimens of calf muscle biopsies were labeled concurrently with antibodies specific for Collagen IV, αSmooth Muscle Actin (αSMA), and endothelial cell CD-31 (Figure 9). Pericytes were identified as α-Smooth Muscle Actin (α-SMA) fluorescence labeling intimately associated with Collagen IV labeling and endothelial cell labeling (CD-31) of micro-vessels (overall diameter < 15 µm) [19].

Because of the very labor-intensive work required for partitioning and quantification of pericyte events in each tissue section, we evaluated pericyte coverage in duplicate slide specimens of calf muscle biopsies from five control subjects, five Stage II patients and five Stage IV patients. Using Quantitative Fluorescence Microscopy, we quantified pericyte coverage as the sum of the areas occupied by pericyte segments per 40× microscopic field. We analyzed five fields of each duplicate slide per control and patient and present the data as medians per group (Figure 9; Fence Box Plot). Medians (M) and interquartile ranges (IQR) were 126 μm^2^, 135μm^2^; 446 μm^2^, 121 μm^2^; and 1077 μm^2^, 564 μm^2^ for control subjects and Stage II and Stage IV PAD patients, respectively. In a randomization test, pericyte coverage of calf muscle micro-vessels of Stage IV patients, was greater than that of control subjects (*p* = 0.006) and Stage II patients (*p* = 0.010). Pericyte coverage in Stage II specimens was greater than that of control specimens (*p* = 0.008). To determine whether differences in coverage across control subjects and PAD patients was due simply to increased micro-vessel density (*cf.* Results, Section 3.4), we determined pericyte coverage as the area of pericyte events per micro-vessel. Coverage per micro-vessel was greater in the calf muscle of Stage IV patients (Median = 16.0 μm^2^; IQR = 11.0) compared to control subjects (Median = 6.0 μm^2^; IQR = 1.5) and Stage II patients (Median = 10.0 μm^2^; IQR = 3.0) at *p* = 0.008 and 0.009, respectively. The difference between control subjects and Stage II patients was significant (*p* = 0.015).

DM, RI and FH were evaluated as covariates in a General Linear Model for fixed factors (Group: Control, Stage II and Stage IV patients). For response variable Pericyte Coverage, the predictive value (adjusted *R*^2^) of the model was 0.745 (*p* < 0.003) where Group (*p* = 0.001) was a significant predictor and DM (*p* = 0.277), RI (*p* = 0.773), and FH (*p* = 0.758) were not significant predictors.

## 4. Discussion

**Figure 10:**
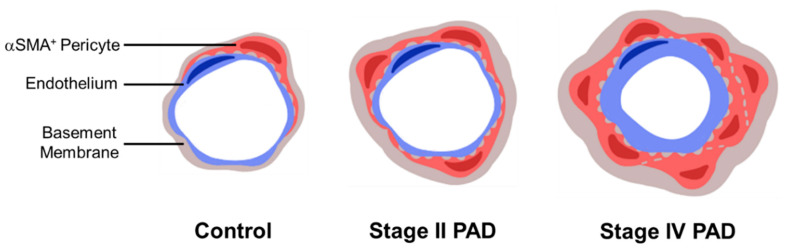
Schematic representation of micro-vessel remodeling in PAD.

The myopathy in the lower extremities of patients with PAD is a consequence of atherosclerotic occlusive disease of the named inflow arteries (macrovascular disease) supplying the legs. This macrovascular disease characteristically affects the aortic bifurcation, the superficial femoral artery at Hunter’s hiatus and the popliteal trifurcation [20]. In the present study, we extend (1) our previous work [15] establishing a pathologic remodeling of micro-vessels (50–150 μm diameter) and collagenous investment of the smallest micro-vessels (≤15 μm diameter) in the calf muscle of PAD patients and (2) the work of Baum et al. [21] demonstrating pathologic thickening of the basement membrane (BM) of terminal micro-vessels in lower leg muscles of PAD patients. We document pathological events in terminal micro-vessels (≤15 μm diameter) of the calf muscle of PAD patients, establishing that vascular pathology associated with the legs of PAD patients extends beyond the atherosclerotic main inflow arteries and affects the entire vascular tree down to the smallest micro-vessels. Pathological events included increased BM thickness, diameter and abundances of Collagen I and Collagen IV. Moreover, pericyte coverage of terminal micro-vessels was increased significantly in both Stage II and Stage IV disease. Generally, micro-vessel pathology was most severe in patients with Stage IV disease (Figure 10). Compared to controls, terminal micro-vessel density in the calf muscle of Stage II and Stage IV patients was increased by 17% and 27%, respectively, suggesting an adaptive response to oxygen and nutrient deficits in the PAD muscle. The nearly universal micro-vessel pathology in the calf muscle specimens suggests that this adaptive response was frustrated.

Micro-vessel architectural changes across stages of PAD are depicted in the cartoon. The number of αSMA^+^ pericytes (red) increases with PAD severity around the endothelium (blue) of micro-vessels less than 15 μm in diameter. The BM (grey) is thickened in association with increased deposition of Collagen I and Collagen IV in the micro-vessels of PAD patients. As the area of αSMA^+^ pericytes increases, the BM becomes thicker and the diameter of the BM annulus increases.

Our work demonstrating increased thickness of the BM of terminal micro-vessels in the calf muscle of patients with PAD confirms the findings of Baum et al. [21]. In this study, we have extended this work by showing coordinated increases in both thickness and diameter of the BM ring of micro-vessels in association with inter-myofiber and microvascular fibrosis in the calf muscle of PAD patients with Stage IV disease. Recent studies have identified pericytes as the principal precursors of myofibroblasts which produce fibrosis in skeletal muscle and other organ systems [22,23]. In phenotype tracing studies, Birbrair et al. [24] identified Type-1 pericytes as fibrogenic in a mouse model of age-dependent skeletal muscle fibrosis and showed that they became fibrogenic myofibroblasts when exposed to TGF-β1. In a study of a mouse model of kidney fibrosis, Wu et al. [25] found that TGF-β1 signaling from damaged tubular epithelial cells activated micro-vessel pericyte-to-myofibroblast transition, causing fibrosis in the kidney. Thus, TGF-β1 production and secretion in a location other than terminal micro-vessels, signals micro-vessel pericytes to produce Collagen I and transition to myofibroblasts which migrate to the ECM and produce organ fibrosis. Expression of both TGF-β1 and Collagen I were shown to increase in a mouse model of chronic kidney disease in response to ischemia/reperfusion [17]. Ischemia/reperfusion is central to the myopathy of the lower legs of PAD patients [26]. In a previous study, we documented fibrotic remodeling of micro-vessels (50 to 150 µm diameter), in the calf muscle of patients with PAD [15]. The collagenous media of these vessels were thickened and contained hyperplastic VSMC that were laden with TGF-β1. In the same study, we noted “collagenous investment” of micro-vessels (≤15 µm diameter). We propose that micro-vessel remodeling documented in the present study is, at least in part, a response to TGF-β1 originating in the hyperplastic VSMC of neighboring, larger micro-vessels. On this basis, we evaluated Collagen I deposition in the terminal micro-vessels of the calf muscle of PAD patients and found a significant increase in Collagen I abundance in the BM of these micro-vessels in patients with Stage IV PAD. Collagen I deposition co-localized with that of Collagen IV, a marker for BM, in terminal micro-vessels. We conclude that the pathology of micro-vessels in PAD patients includes a BM fibrosis at the level of terminal micro-vessels and is likely mediated by TGF-β1 activated pericytes. We anticipate that these changes in the BM of terminal micro-vessels have an adverse effect on micro-vessel compliance, blood flow and nutrient and oxygen diffusion.

The same features of vascular remodeling we have documented for the calf muscle of patients with PAD [15] have been documented for Pulmonary Arterial Hypertension (PAH) [27,28]. Both pathologies are linked to tissue hypoxia and are characterized by substantial thickening of vascular media caused by extensive proliferation and hypertrophy of VSMC that exhibit increased expression of TGF-β1, and deposition of extracellular matrix; features that extend to normally non-muscularized microvasculature. In a more recent study of PAH, Ricard et al. [29] documented abnormally increased pericyte coverage of micro-vessels, attributing this to pericyte proliferation induced by Fibroblast Growth Factor-2 produced and secreted by dysfunctional endothelial cells. TGF-β1 promoted pericyte transitioning to “smooth muscle-like cells” in agreement with Wu et al. [25] and Birbrair et al. [24] who showed that TGF-β1 stimulated pericyte-myofibroblast transitioning but did not stimulate pericyte proliferation. In the present study, we document increased pericyte coverage of terminal micro-vessels in the calf muscle of PAD patients. The greatest increase occurred in the calf muscle of patients with Stage IV PAD. We propose that fibrotic deposition of Collagen I in the BM of terminal micro-vessels is a response to TGF-β1 signaling from the hyperplastic VSMC of neighboring, larger micro-vessels. Future studies are needed to determine whether endothelial cells are damaged in the calf muscle of PAD patients. However, our study of oxidative damage in the calf muscle of PAD patients identified abnormal accumulation of carbonyl adducts in the micro-vessels of the calf muscle in these patients (Weiss et al. 2013, Figure 1) [14]. Carbonyl adducts may contribute to quenching of endothelial cell nitric oxide, producing endothelial cell dysfunction that drives pericyte-mediated fibrosis of the BM and the ECM [30].

The architectural changes observed in this study are consistent with reports of perfusion deficits in PAD patients. Several studies, using contrast-enhanced ultrasound [31], have documented a delayed time to peak microvascular blood flow in PAD patients relative to controls after exercise [32,33,34] and post-occlusive reactive hyperemia [35]. During moderate contractile exercise, microvascular blood volume was slightly lower than controls, whereas microvascular blood velocity was substantially diminished in PAD patients. Furthermore, Duerschmied et al. [33] demonstrated, using contrast-enhanced ultrasound, that blood traversed the muscle more slowly in PAD patients compared to controls, with the greatest delays in contrast transit time arising within the microvascular compartment, which is consistent with our observation of increased BM thickness and diameter of terminal micro-vessel in association with increased pericyte coverage of these micro-vessels. The BM thickening of PAD micro-vessels described in our study may contribute directly to the slowing of the microcirculation by decreasing compliance of the terminal micro-vessels.

A limitation of this study is the relatively small number of patients analyzed. Although we found no significant contribution of DM, FH, and RI to microvascular pathology, the small number of patients per group limited the statistical power of the analysis. Micro-vessel thickening has been observed in the microvascular beds of several organs in patients with DM and patients with RI. However, in patients analyzed in this study, BM thickening and increased diameter, increased pericyte coverage of micro-vessels, microvascular fibrosis, and myofibrosis did not differ between patients with and without DM or RI, which suggests that PAD alone is enough to induce and propagate microvascular pathology. DM microangiopathy is well characterized in several organs. Death of pericytes, and consequently, microvascular instability and decreased capillary density are hallmark features of diabetic microvascular complications and are well documented features of diabetic retinopathy [36]. Our observations that microvascular density and pericyte coverage are increased in PAD patients with and without DM suggests that the microangiopathy of PAD is different from, and likely dominant to the microangiopathy observed in DM. Additional studies are necessary to better characterize the contribution of insulin resistance and DM to PAD microvascular pathology.

Regarding the microvascular disease several, questions remain. Future research needs to address the contribution of oxidative damage to micro-vessel pathology, including BM fibrosis and increased pericyte coverage. Understanding the relationship between microvascular pathology and micro-perfusion offers a unique opportunity for the development of non-invasive diagnostic evaluation of PAD limbs and may have great utility in the assessment of the efficacy of different therapies. Furthermore, non-invasive imaging modalities, such as contrast-enhanced ultrasonography [31,32,35] and magnetic resonance imaging [37,38], could enhance our understanding of the functional alterations of the microvasculature of PAD limbs. Understanding PAD microvascular pathology may offer opportunities for more individualized therapies, which may include angiotensin-inhibitors, anti-hypertensives, statins, and DM medications, and may aid in the identification of patients who are likely to experience significant disease progression and need more aggressive therapy.

## 5. Conclusions

Our study establishes the extension of vascular pathology of PAD beyond macrovascular atherosclerosis to pathologic fibrosis and remodeling of the smallest micro-vessels in ischemic muscles of the lower legs of PAD patients. The microvascular disease of PAD is characterized by increased thickness and diameter of the BM in association with increased Collagen I deposition, an index of fibrosis, in the BM, and increased pericyte coverage of the terminal micro-vessels. Fibrosis of the BM likely is driven, at least in part, by TGF-β1 signaling from hyperplastic VSMC in neighboring, larger micro-vessels, which promotes Collagen I production by pericytes and their transitioning to myofibroblasts. In addition, oxidative damage (which is central to the myopathy of PAD) may serve as a central mechanism of endothelial cell damage, and subsequent signaling that drives pericyte proliferation and increased pericyte coverage of the terminal micro-vessels. Our findings provide insight into the pathophysiology of PAD and direction for future mechanistic studies, and consequently, a basis for improved diagnosis and treatment for patients with PAD.

## Figures and Tables

**Table 1:** Patient demographics.

	Control	Stage II PAD	Stage IV PAD
Number of Subjects	14	15	16
Mean Age (years)	67.3 ± 1.3	64.3 ± 1.2	69.4 ± 1.0
Ankle Brachial Index	1.09 ± 0.03	0.60 ± 0.03 **	0.18 ± 0.06 **^,^^ǂ^
Gender (male/female)	14/0	15/0	15/1
Smoking (never/current/former)	6/6/2	0/6/9	2/9/5
Coronary artery disease	4/14	3/15	8/16
Cerebrovascular disease (none/TIA ^A^/stroke)	13/1/0	13/0/2	12/1/3
Obesity ^B^	6	3	4
Dyslipidemia	10	8	7
Diabetes Mellitus	2	2	8 *^,^^†^
Chronic Obstructive Pulmonary Disease	0	3	1
Renal insufficiency ^C^	1	0	7 *^,^^ǂ^
Statins	9	12	9
Family history of cardiovascular disease	5	7	14 *^,^^†^
Hypertension	8	9	14

Continuous variables are presented as mean ± standard error of the mean and were analyzed with the Kruskal-Wallis test. Categorical variables were analyzed with the Yates’ Continuity Corrected Chi-square test. Significantly different from control at *p* < 0.05 * or *p* < 0.005 **. Significantly different from Stage II PAD at *p* < 0.05 ^†^ or *p* < 0.005 ^ǂ^. ^A^ Transient Ischemic Attack. ^B^ Body Mass Index > 30 kg/m^2^. ^C^ Creatinine Clearance < 60 mL/min/1.73 m^2^.

**Table 2:** Measurements of the basement membrane of micro-vessels in the calf muscle of control subjects and patients with Stage II and Stage IV peripheral artery disease (PAD).

Micro-Vessel BM Measurements (μm)	Control	Stage II PAD	Stage IV PAD
Thickness	1.408 ± 0.025	1.577 ± 0.030 *	1.747 ± 0.060 *^,^^†^
Inner Diameter	3.243 ± 0.094	3.295 ± 0.064	3.972 ± 0.124 *^,^^ǂ^
Overall Diameter	6.058 ± 0.111	6.448 ± 0.112	7.466 ± 0.224 *^,^^ǂ^

Calf muscle biopsy specimens were labeled with anti-Collagen IV antibody and micro-vessel BM architectural features were measured. Measurements were obtained for approximately 50 to 200 micro-vessels per biopsy specimen. Control (*N* = 14), PAD Stage II (*N* = 15), and PAD Stage IV (*N* = 16) measurements are presented as mean ± S.E.M. Data were analyzed by Student’s *t*-test. * Significantly different from Control (*p* < 0.001). ^†^ Significantly different from Stage II PAD (*p* = 0.02). ^‡^ Significantly different from Stage II PAD (*p* < 0.001).
